# Application of Epigenetic biomarkers in the precision medicine for sepsis-induced acute lung injury: From risk stratification to targeted therapy

**DOI:** 10.1016/j.isci.2026.116771

**Published:** 2026-07-16

**Authors:** Jingyan Wei, Ruliu Xiong, Tian Zhang, Fengao Liang, Jianfeng Chen, Yanhong Zhao, Ying Huang, Zhenyan Huang, Chenwen Peng

**Affiliations:** 1 Zhongshan Hospital of Traditional Chinese Medicine Affiliated to Guangzhou University of Traditional Chinese Medicine, Zhongshan 528400, China; 2 Zhongshan Hospital of Traditional Chinese Medicine, Zhongshan 528400, China

**Keywords:** health sciences, medicine, pathophysiology, respiratory medicine

## Abstract

Sepsis-induced acute lung injury (SI-ALI) remains a leading cause of mortality in critical care, characterized by complex pathophysiology and substantial patient heterogeneity that challenge conventional diagnostic and therapeutic approaches. Precision medicine, which tailors prevention, diagnosis, and treatment strategies to individual patient characteristics, offers a promising avenue to improve outcomes in this condition. This review focuses on the pivotal role of epigenetic modifications—including DNA methylation, histone modifications, and non-coding RNAs—in the pathogenesis and progression of SI-ALI. We comprehensively summarize recent advances in understanding how these epigenetic mechanisms contribute to disease heterogeneity and influence clinical trajectories. Furthermore, we highlight the emerging potential of epigenetic biomarkers as tools for risk stratification, prognostic evaluation, and guidance of targeted therapeutic interventions in SI-ALI. By integrating current research findings, this article aims to establish a novel theoretical framework and translational direction for the precision management of SI-ALI, ultimately facilitating the development of personalized treatment strategies to address the unmet clinical needs in this critical condition.

## Graphical abstract

**Figure:**
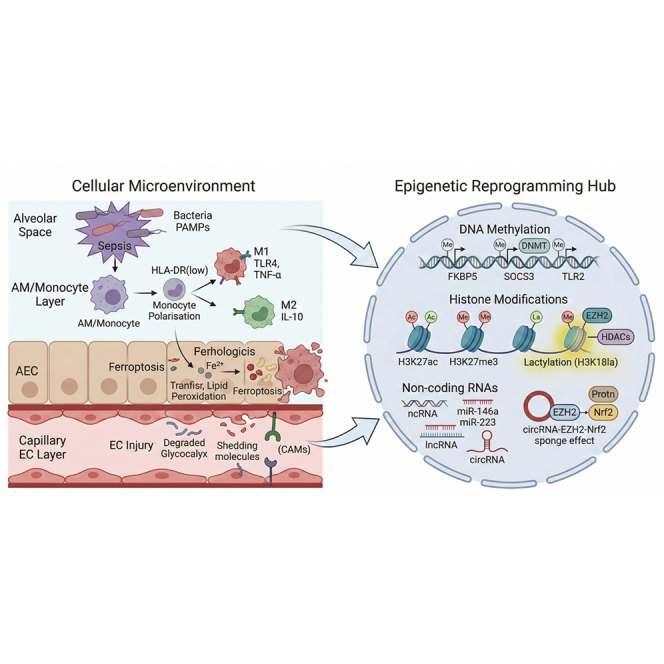

## Introduction

Sepsis is a life-threatening condition characterized by a dysregulated host response to infection, resulting in organ dysfunction and substantial global mortality. Among affected organs, the lung is particularly vulnerable, with sepsis frequently precipitating acute lung injury (ALI) or its more severe form, acute respiratory distress syndrome (ARDS).^1^ The pathogenesis of sepsis-induced ALI/ARDS (SI-ALI/ARDS) is complex and multifactorial, involving systemic inflammation, immune dysregulation, oxidative stress, endothelial dysfunction, and disruption of the alveolar-capillary barrier.^2^^,^^3^ Despite advances in supportive care, including lung-protective ventilation strategies, mortality rates remain unacceptably high, and effective targeted therapies are still lacking.^4^^,^^5^ This clinical challenge is further compounded by the marked heterogeneity of SI-ALI/ARDS in terms of etiology, host response, and disease trajectory, which collectively limits the efficacy of conventional one-size-fits-all treatment approaches.^6^^,^^7^

Current diagnostic and therapeutic strategies for SI-ALI/ARDS primarily rely on clinical and physiological parameters—such as the PaO2/FiO2 ratio and chest imaging—which lack specificity and fail to capture the underlying molecular heterogeneity.^8^^,^^9^ Moreover, clinical phenotypes are influenced by the source of sepsis (pulmonary vs. extrapulmonary), microbial factors, and host immune status, leading to distinct pathophysiological mechanisms and treatment responses.^10^^,^^11^ This heterogeneity underscores an urgent need for biomarkers that enable risk stratification, early diagnosis, and personalized therapeutic interventions.

Epigenetics, defined as heritable changes in gene expression without alterations in the DNA sequence, has emerged as a critical regulatory layer linking environmental stimuli—including pathogen exposure and inflammatory signals—to gene expression programs in immune and structural lung cells.^12^ Key epigenetic mechanisms include DNA methylation, histone modifications, chromatin remodeling, and non-coding RNAs, all of which dynamically modulate chromatin accessibility and transcriptional activity.^13^ Importantly, these modifications are reversible and sensitive to environmental cues, positioning them as attractive candidates for both biomarkers and therapeutic targets in SI-ALI/ARDS.^14^^,^^15^

Recent studies have demonstrated that epigenetic alterations contribute to the dysregulated immune and inflammatory responses in sepsis and SI-ALI/ARDS. For instance, aberrant DNA methylation patterns and histone modifications have been linked to the regulation of pro-inflammatory cytokines, immune cell polarization, and endothelial barrier integrity.^16^ Non-coding RNAs, including microRNAs and long non-coding RNAs (lncRNAs), modulate key signaling pathways such as NF-κB activation and NLRP3 inflammasome signaling, thereby influencing the magnitude and resolution of inflammation.^17^ Furthermore, epigenetic reprogramming of immune cells during sepsis shapes the trajectory of immune dysfunction—from hyperinflammation to immunosuppression—ultimately affecting patient outcomes.^18^^,^^19^

The dynamic and tissue-specific nature of epigenetic modifications offers the potential to identify distinct molecular endotypes within the heterogeneous SI-ALI/ARDS population. Integrative multi-omics approaches, combining epigenomic, transcriptomic, proteomic, and metabolomic data, have begun to unravel these endotypes, revealing differential immune activation states, metabolic reprogramming, and epigenetic signatures associated with disease severity and prognosis.^20^^,^^21^ Such stratification could inform precision medicine strategies, enabling tailored interventions that target specific epigenetic regulators or pathways.^14^^,^^22^

Beyond their biomarker potential, epigenetic modifications represent promising therapeutic targets. Pharmacological agents that modulate DNA methylation and histone acetylation—such as DNA methyltransferase inhibitors (DNMTis) and histone deacetylase inhibitors (HDACis)—have demonstrated efficacy in preclinical models of sepsis and ALI by restoring immune homeostasis and reducing inflammation.^23^^,^^24^ Novel approaches, including epigenetic editing and nanotechnology-based delivery systems, are being explored to enhance specificity and minimize off-target effects.^25^^,^^26^ Moreover, understanding the interplay between metabolism, oxidative stress, and epigenetic regulation may yield combinatorial therapies that address the multifaceted pathophysiology of SI-ALI/ARDS.^27^

Despite these advances, significant challenges remain in translating epigenetic biomarkers and therapies into clinical practice. These include the need for standardized methodologies, validation in large and diverse patient cohorts, and integration with clinical parameters to improve predictive accuracy.^28^^,^^29^ Additionally, the temporal dynamics of epigenetic changes during sepsis necessitate longitudinal monitoring to capture disease progression and therapeutic response.^18^ Addressing these challenges will require interdisciplinary collaboration and the development of point-of-care assays suitable for critical care settings.^30^^,^^31^ This review aims to comprehensively discuss the current understanding of epigenetic regulation in SI-ALI/ARDS, highlight emerging biomarkers and therapeutic strategies, and outline future directions to overcome existing challenges—ultimately realizing the full potential of epigenetics in precision medicine for sepsis-induced lung injury.

## Methods

### Search strategy

This narrative review was conducted following the PRISMA (Preferred Reporting Items for Systematic Reviews and Meta-Analyses) guidelines to ensure transparency. We systematically searched PubMed, Web of Science, and Scopus for literature published from January 2020 to the present. The search strategy combined MeSH terms and free-text keywords across three core domains using Boolean operators. The first domain, Disease Context, included terms such as “Sepsis,” “Sepsis-Induced Acute Lung Injury (SI-ALI),” “Acute Respiratory Distress Syndrome (ARDS),” “Sepsis-associated ARDS.” The second domain, Epigenetic Mechanisms, encompassed “Epigenetics,” “DNA methylation,” “Histone modification,” “Histone acetylation,” “Histone methylation,” “Histone lactylation,” “Non-coding RNA,” “microRNA,” “lncRNA,” “circular RNA,” and “RNA methylation.” The third domain, Biomarkers and Therapeutics, covered “Epigenetic biomarkers,” “Risk stratification,” “Prognostic prediction,” “Precision medicine,” “Targeted therapy,” “Epigenetic drugs,” “DNMT inhibitors,” “HDAC inhibitors,” “miRNA mimics,” “AntagomiR,” “Multi-omics,” and “Machine learning.” Terms within each domain were connected using the Boolean operator OR, while the three domains were combined using the Boolean operator AND to maximize sensitivity and relevance. Additionally, reference lists of key articles and recent reviews were manually screened to identify relevant studies missed by the database search.

### Selection criteria

Inclusion criteria were: (1) original research, including *in vitro*, *in vivo*, or clinical studies, focusing on epigenetic modifications or epigenetic biomarkers in sepsis, ALI, or ARDS; (2) studies investigating non-coding RNAs, DNA methylation, histone modifications, or epigenetic therapeutics in the context of sepsis-induced lung injury; (3) articles published in English; and (4) studies providing clear mechanistic insights or clinical data on risk stratification, prognosis, or targeted therapy. Exclusion criteria included: (1) studies that focused exclusively on infectious or non-sepsis-related ALI without addressing epigenetic mechanisms; (2) types of non-original research, such as editorials and conference abstracts; and (3) articles lacking full-text availability or methodological clarity.

### Screening results

**Figure 1:**
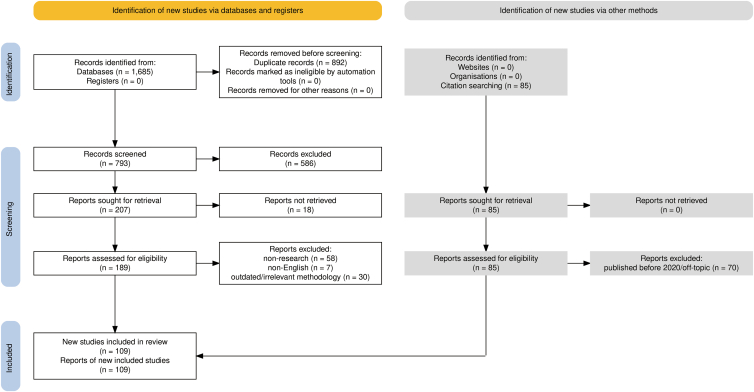
PRISMA flowchart for literature search and study selection A total of 1,685 records were identified from database searches (PubMed, Web of Science, and Scopus). After duplicate removal (*n* = 892), 793 records were screened, and 586 were excluded based on titles and abstracts. Of the 207 full-text articles assessed, 18 were inaccessible, and 95 were excluded (58 non-research articles, 7 non-English studies, and 30 with outdated or irrelevant methodology). Citation tracking identified 85 additional records, of which 70 were excluded, yielding 15 eligible reports. Ultimately, 109 studies were included in this review.

This study followed the PRISMA framework for literature screening (Figure 1).^32^ Two authors independently conducted database searches across PubMed, Web of Science, and Scopus using the described strategy. The initial combined search retrieved 1,685 records. After removing 892 duplicates using an automated reference manager (EndNote ×20), 793 unique records remained for initial screening. The titles and abstracts of these records were screened against the selection criteria. This process led to the exclusion of 586 articles that were unrelated to the research topic. The remaining 207 articles proceeded to full-text retrieval. Of these, 18 articles were inaccessible and were excluded. A detailed eligibility assessment was then carried out on the remaining 189 articles. This led to the removal of 58 non-research articles, 7 non-English studies, and 30 studies excluded due to outdated findings or insufficient methodological relevance to the review’s focus on epigenetic regulation in sepsis-induced ALI. Subsequently, citation tracking from the reference lists of key included articles and recent major reviews was conducted. This process identified 85 potentially relevant articles not captured by the initial database search. After evaluation, 70 of these were excluded as they were published before 2020 or were unrelated to the core mechanistic focus of this review. The remaining 15 articles met the inclusion criteria and were included in the review.

Ultimately, a total of 109 studies that met all inclusion criteria were included in this narrative review. The final body of literature provides a foundation for discussing epigenetic biomarkers and targeted therapies in SI-ALI, encompassing both direct and highly supportive evidence.

## Pathophysiology and clinical heterogeneity of sepsis-induced acute lung injury

### Complex pathogenesis and molecular endotypes of sepsis-induced acute lung injury

SI-ALI represents a multidimensional pathological state intricately linked to a cascade of interconnected processes, including dysregulated systemic inflammation, disruption of the vascular endothelial and alveolar epithelial barriers, coagulation abnormalities, and immune dysfunction. The initial septic insult triggers a dysregulated host immune response, leading to the massive release of pro-inflammatory cytokines such as tumor necrosis factor-alpha (TNF-α) and interleukins (IL-1β, IL-6). This cytokine storm subsequently disrupts the alveolar-capillary barrier, resulting in increased vascular permeability, pulmonary edema, and impaired gas exchange—hallmark features of ALI and its more severe form, ARDS.^33^ Vascular endothelial injury can further activate coagulation pathways, promoting microvascular thrombosis in the pulmonary circulation. This process exhibits a dualistic role: Moderate pulmonary thrombosis may confer protection through endothelial Alox15-mediated lipid signaling, whereas severe thrombosis exacerbates lung injury.^34^ Concurrently, the accompanying immunosuppression impairs the phagocytic function of neutrophils and macrophages, increasing the risk of secondary infections and adversely affecting patient outcomes.^35^^,^^36^ A major challenge in the clinical management of SI-ALI lies in the marked inter-patient heterogeneity, manifesting as distinct inflammatory endotypes, immune profiles, and clinical trajectories. Patients with a hyperinflammatory endotype exhibit elevated pro-inflammatory cytokine levels, greater vasopressor requirements, and higher mortality rates, whereas those with a hypoinflammatory endotype display opposite characteristics. This dichotomy underscores the pressing need for personalized therapeutic approaches.^37^

From a cellular and organ regulatory perspective, multiple mechanisms contribute to the pathogenesis and progression of SI-ALI. At the cellular level, the polarization state of macrophages plays a pivotal role: classically activated M1 macrophages exacerbate inflammatory responses and tissue damage through the secretion of pro-inflammatory mediators, whereas alternatively activated M2 macrophages mediate inflammation resolution and tissue repair. An imbalance favoring M1 polarization aggravates lung injury.^38^^,^^39^ This process is intricately regulated by multiple signaling pathways, including those mediated by Notch1/β-catenin/nuclear factor-κB, cyclic GMP-AMP synthase/mammalian target of rapamycin complex 1, and Toll-like receptor (TLR) 7; additionally, dysregulation of mitochondrial dynamics in alveolar macrophages influences their polarization state.^40^^,^^41^^,^^42^ Neutrophil heterogeneity also contributes to pathogenesis, with subpopulations expressing p75NTR and CD64 exhibiting pathogenic features characterized by impaired phagocytosis and increased cytokine secretion. Concurrently, neutrophil extracellular traps (NETs) released during sepsis amplify the inflammatory cascade, further exacerbating lung injury.^43^ Notably, strategies targeting CXCR4 signaling—such as PTP1B inhibitors—to promote a senescent neutrophil phenotype have demonstrated therapeutic potential in preclinical models.^44^ Furthermore, the gut-lung axis represents another critical regulatory dimension in SI-ALI pathogenesis. Sepsis-induced disruption of the intestinal barrier and alterations in gut microbiota lead to the translocation of microbial products, triggering systemic inflammation and consequently aggravating lung injury. This bidirectional crosstalk between the gut and lung involves complex immune and microbial interactions, offering novel therapeutic directions.^45^^,^^46^

**Figure 2:**
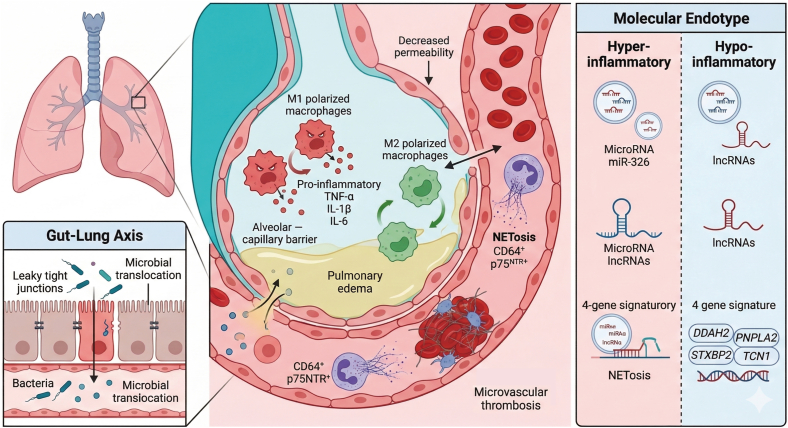
Pathogenesis and molecular endotypes of SI-ALI This figure illustrates the key components involved in SI-ALI pathogenesis and molecular endotypes. The gut-lung axis contributes to lung injury through leaky tight junctions, microbiota translocation, and microbial translocation. Molecular endotypes are categorized into hyper-inflammatory and hypo-inflammatory subtypes, characterized by M1 versus M2 macrophage polarization, pro-inflammatory cytokines (TNF-α, IL-1β, IL-6), NETosis, CD64^+^ p75NTR^+^ neutrophil subpopulations, and non-coding RNAs (miR-326, lncRNAs). A four-gene signature comprising DDAH2, PNPLA2, STXBP2, and TCN1 is associated with disease endotypes. Microvascular thrombosis represents an additional pathophysiological process in pulmonary circulation.

Molecular regulatory mechanisms and phenotypic identification provide critical support for the precision management of SI-ALI, as shown in Figure 2. Non-coding RNAs, including microRNAs and lncRNAs carried by extracellular vesicles, regulate gene expression networks associated with inflammation, apoptosis, and barrier function in SI-ALI. For instance, miR-326 suppresses TLR4-mediated inflammation, while interference with lncRNA-p21 alleviates lung injury.^47^^,^^48^ Epigenetic regulators such as MEGF6 and Rev-Erbα modulate inflammatory and oxidative stress pathways, positioning them as promising therapeutic targets.^49^^,^^50^ Biomarkers including surfactant protein D (SP-D) have been correlated with disease severity and prognosis in pediatric ARDS, underscoring the potential clinical utility of such markers.^51^^,^^52^ Furthermore, molecular phenotyping can guide clinical treatment decisions, as evidenced by differential responses to activated protein C among distinct sepsis phenotypes. Therefore, a comprehensive understanding of SI-ALI pathogenesis and precise identification of molecular endotypes are essential for developing targeted therapies and improving patient outcomes.

### Current limitations in diagnosis and treatment and the landscape of biomarkers

The current clinical diagnosis and management of SI-ALI face significant challenges, primarily stemming from inherent limitations in existing diagnostic criteria and biomarker applications. The Berlin definition, serving as the cornerstone for diagnosing ARDS—including SI-ALI—relies predominantly on physiological parameters such as the PaO2/FiO2 ratio, imaging findings, and timing of onset. While these criteria provide a standardized framework, they fail to capture the underlying molecular and pathophysiological heterogeneity of SI-ALI. Reliance solely on physiological indicators leads to delayed recognition of disease onset and progression, consequently impeding timely and targeted therapeutic interventions. The complex interplay of systemic inflammation, immune dysregulation, and organ crosstalk in sepsis-induced lung injury is not reflected in these clinical parameters, limiting their prognostic and therapeutic guidance value.^33^^,^^53^

Traditional protein biomarkers, including procalcitonin (PCT) and C-reactive protein (CRP), have been extensively employed to aid diagnosis and monitor sepsis and its complications, including ALI. However, these markers lack specificity for SI-ALI and fail to distinguish between different pathophysiological subtypes or endotypes of the disease. For instance, elevated PCT and CRP levels indicate systemic inflammation but do not reveal the molecular pathways driving lung injury or the severity of alveolar-capillary barrier disruption. Furthermore, these biomarkers cannot reliably predict patient outcomes or therapeutic responses, restricting their utility in precision medicine approaches.^54^^,^^55^ Recent research has highlighted the heterogeneity of SI-ALI, with distinct molecular signatures related to endothelial activation, immune cell infiltration, and regulated cell death pathways such as ferroptosis and pyroptosis.^56^ Yet, current protein biomarkers fail to capture these nuanced biological processes, underscoring the need for more specific and mechanistically informative markers.^57^ There is an urgent clinical need for novel molecular biomarkers capable of early identification of high-risk patients, stratifying SI-ALI into distinct pathophysiological subtypes, and guiding targeted therapeutic interventions. Emerging evidence suggests that epigenetic markers, non-coding RNAs—including microRNAs and lncRNAs—and extracellular vesicle-derived molecules hold promise in fulfilling these roles. For example, microRNAs such as miR-326 have been shown to modulate inflammatory signaling pathways like TLR4, thereby influencing lung injury severity and exhibiting potential as both biomarkers and therapeutic targets.^48^ Similarly, lncRNAs have been implicated in regulating immune responses and apoptosis in sepsis, with some demonstrating potential as prognostic indicators and intervention points.^58^^,^^59^

Furthermore, the identification of specific protein biomarkers associated with endothelial dysfunction—such as angiopoietin-2, intercellular adhesion molecule-1 (ICAM-1), and Tie2—has advanced our understanding of SI-ALI pathogenesis and may aid in distinguishing indirect lung injury subtypes associated with sepsis.^60^ However, these markers remain insufficient for providing comprehensive risk stratification or guiding precision therapeutics. Advances in multi-omics approaches and machine learning have facilitated the discovery of novel biomarker panels integrating genetic, transcriptomic, and proteomic data, which demonstrate enhanced diagnostic and prognostic accuracy. For instance, a recent study identified a four-gene signature (DDAH2, PNPLA2, STXBP2, TCN1) with high diagnostic performance for sepsis-induced ALI through bioinformatics analysis and experimental validation, highlighting the potential of integrated molecular profiling.^61^ Beyond molecular biomarkers, functional imaging and novel probes targeting specific pathophysiological processes—such as pulmonary hypoxia and nitroreductase activity—have been developed to monitor disease progression and therapeutic responses with greater precision.^62^ These tools complement molecular markers by providing spatial and temporal resolution of lung injury dynamics.

Despite these advances, significant barriers remain in translating novel biomarkers into routine clinical practice. Challenges include the need for standardized, rapid, and cost-effective assays; validation in large and diverse patient cohorts; and integration into clinical decision-making algorithms. Moreover, the heterogeneity of sepsis and SI-ALI necessitates biomarker panels capable of capturing the complexity of immune dysregulation, organ crosstalk, and regulated cell death pathways, rather than reliance on single markers.^63^ Consequently, emerging epigenetic markers, non-coding RNAs, extracellular vesicle contents, and multi-omics-derived gene signatures offer promising avenues for overcoming these limitations and advancing precision medicine in SI-ALI.

## Epigenetic fundamentals and their regulatory roles in sepsis and ALI

### Overview of major epigenetic mechanisms

Epigenetic regulation encompasses a diverse array of molecular modifications that influence gene expression without altering the DNA sequence, playing a pivotal role in the pathogenesis of sepsis-induced ALI. Among these mechanisms, DNA methylation, histone modifications, and non-coding RNAs are principal contributors to the dynamic regulation of gene activity in response to environmental and pathological stimuli such as sepsis. DNA methylation typically involves the addition of methyl groups to cytosine residues, commonly within CpG islands in gene promoter regions, resulting in transcriptional repression. In the context of sepsis and ALI, aberrant DNA methylation patterns have been observed, particularly hypomethylation of pro-inflammatory gene promoters, leading to their overexpression and exacerbated inflammatory responses. For instance, studies have demonstrated that DNA methyltransferase 1 (DNMT1) activity regulates inflammatory gene expression through methylation changes, and its interaction with lncRNAs influences microRNA expression, thereby affecting macrophage polarization and inflammatory cascades in sepsis-associated ALI.^64^ This epigenetic plasticity enables rapid adaptation of immune cells to the septic environment but also contributes to inflammatory dysregulation and tissue injury.

Histone modifications constitute another critical layer of epigenetic control, involving post-translational modifications of histone tails—such as acetylation, methylation, and phosphorylation—that alter chromatin structure and accessibility. For example, acetylation of histone H3 at lysine 27 (H3K27ac) is typically associated with transcriptional activation, relaxing chromatin and facilitating transcription factor binding. In sepsis-induced ALI, increased H3K27 acetylation has been linked to enhanced expression of autophagy-related genes that attenuate oxidative stress and pyroptosis in macrophages, thereby alleviating lung injury.^65^ Conversely, histone methylation mediated by enhancer of zeste homolog 2 (EZH2), such as trimethylation of H3K27 (H3K27me3), generally leads to gene silencing. Research indicates that EZH2 promotes ferroptosis in alveolar epithelial cells by increasing H3K27me3 at the promoter of USP10—a deubiquitinase that stabilizes the antioxidant enzyme GPX4—thereby exacerbating lung injury in sepsis.^66^ Furthermore, EZH2 influences macrophage polarization by modulating histone methylation patterns, altering the balance between pro-inflammatory M1 and anti-inflammatory M2 phenotypes, which is crucial in the progression of sepsis and ALI.^67^ These findings underscore the duality of histone modifications in regulating inflammatory and cell death pathways during sepsis.

Non-coding RNAs, particularly miRNAs and lncRNAs, represent a versatile class of epigenetic regulators that modulate gene expression through post-transcriptional regulation or chromatin remodeling. miRNAs can bind target mRNAs to inhibit translation or promote degradation, thereby fine-tuning inflammatory signaling networks. For instance, the miR-495 axis has been implicated in regulating oxidative stress and pyroptosis in sepsis-associated ALI, with its expression subject to epigenetic regulation by lncRNA SNHG1 and DNMT1, highlighting a complex regulatory circuit influencing macrophage function and lung tissue integrity.^64^ Additionally, circular RNAs (circRNAs) such as circEXOC5 have been found to promote pyroptosis by recruiting EZH2 to suppress Nrf2, a key antioxidant transcription factor, thereby exacerbating inflammation and cell death in septic lungs.^68^ Moreover, circMAPK1 regulates macrophage pyroptosis by destabilizing KDM2B mRNA, which epigenetically modulates WNK1 expression and the NLRP3 inflammasome pathway, illustrating the intricate interplay between non-coding RNAs and histone modifications in sepsis-induced lung injury.^69^

Emerging evidence also highlights the role of RNA modifications, such as m6A methylation, in sepsis. m6A represents the most prevalent internal modification in eukaryotic mRNA and is dynamically regulated by writer, eraser, and reader proteins. Dysregulation of m6A methylation has been associated with altered inflammatory responses and organ dysfunction in sepsis, including ALI. Studies suggest that m6A modifications influence immune cell infiltration, cytokine expression, and transcript stability relevant to lung injury, indicating that targeting m6A machinery may offer novel therapeutic avenues.^70^^,^^71^

Beyond these classical epigenetic mechanisms, metabolic-epigenetic crosstalk has garnered increasing attention in sepsis research. Lactate, a metabolic byproduct elevated during sepsis, can induce histone lactylation—a novel epigenetic mark that modifies histone lysine residues and alters gene transcription. For instance, lactylation of histone H3 at lysine 18 (H3K18la) and of the transcription factor early growth response factor 1 (EGR1) promotes endothelial glycocalyx degradation, a critical event in the pathogenesis of sepsis-induced ALI.^72^ The RBM25-Acly axis has been identified as a key regulator linking metabolic changes to histone lactylation and transcriptional reprogramming, providing a mechanistic link between altered metabolism and epigenetic regulation in sepsis.^73^

### Central role of epigenetics in the pathogenesis of sepsis-induced acute lung injury

SI-ALI manifests as a complex interplay between immune dysregulation and tissue injury, wherein epigenetic mechanisms have emerged as critical regulators of disease progression. Pathogen-associated molecular patterns (PAMPs) and damage-associated molecular patterns (DAMPs) activate innate immune receptors such as TLRs, initiating signaling cascades that profoundly reshape the epigenetic landscape of immune cells—including macrophages and neutrophils—as well as pulmonary parenchymal cells. These epigenetic alterations encompass DNA methylation, histone modifications, and non-coding RNA regulation, which coordinately modulate gene expression without altering the DNA sequence. For instance, the histone methyltransferase EZH2 has been shown to mediate H3K27me3 at promoter regions of key regulatory genes, thereby suppressing protective genes and promoting ferroptosis in alveolar epithelial cells, exacerbating lung injury in sepsis models.^66^ Similarly, the circRNA circEXOC5 recruits EZH2 to epigenetically suppress the antioxidant transcription factor Nrf2, promoting pyroptosis in pulmonary microvascular endothelial cells and release of inflammatory cytokines, further amplifying lung injury.^68^ These findings illustrate how PAMPs and DAMPs-driven epigenetic reprogramming directly influences both immune and structural lung cells, thereby orchestrating the pathological environment of SI-ALI.

This epigenetic remodeling leads to sustained activation of pro-inflammatory genes—including TNF-α, IL-1β, and IL-6—which are central mediators of the cytokine storm characteristic of sepsis and SI-ALI. Epigenetic modifications at these loci, encompassing alterations in histone acetylation and methylation status, maintain their transcriptional upregulation, thereby perpetuating inflammation and tissue injury. For example, inhibition of HDAC3 has been demonstrated to increase H3K27ac levels, enhance expression of autophagy-related genes, and reduce oxidative stress and pyroptosis in macrophages, consequently alleviating lung injury.^65^ Furthermore, metabolic-epigenetic crosstalk, such as lactate-induced histone lactylation, alters chromatin accessibility and gene transcription in endothelial cells, promoting expression of genes like EGR1 that drive glycocalyx degradation and vascular dysfunction—processes implicated in SI-ALI pathogenesis.^73^ These epigenetic changes not only amplify inflammatory cascades but also disrupt pulmonary barrier integrity and cellular homeostasis, exacerbating the severity of lung injury.

Concurrently, epigenetic mechanisms contribute to the immunosuppressive phase of sepsis, often termed immune paralysis, which increases susceptibility to secondary infections and worsens prognosis. This is exemplified by epigenetic suppression of antigen presentation machinery, including downregulation of HLA-DR expression on monocytes and macrophages mediated by histone modifications and DNA methylation alterations. Such suppression impairs effective antigen presentation and adaptive immune activation, thereby fostering an immunosuppressive environment.^74^ The dynamic nature of epigenetic regulation enables the transition from hyperinflammation to immune exhaustion, highlighting its central role in the biphasic immune response characteristic of sepsis. Moreover, epigenetic regulators such as m6A RNA methylation have been implicated in modulating inflammatory gene expression and organ dysfunction during sepsis, including ALI, by controlling RNA stability and translational efficiency.^75^ These modifications finely tune immune responses and may serve as targets for restoring immune function.

Macrophage polarization, a critical determinant of the inflammatory milieu in SI-ALI, is likewise tightly controlled by epigenetic mechanisms. The balance between pro-inflammatory M1-like and anti-inflammatory M2-like macrophages is influenced by histone modifications and non-coding RNAs that regulate expression of transcription factors and cytokine genes. For instance, inhibition of EZH2 shifts macrophage polarization toward an M2 phenotype, thereby reducing inflammation and fibrosis in lung injury models.^76^ Additionally, endothelial cell-derived chemokines such as CCL7 induce metabolic and epigenetic reprogramming in CCR1-positive macrophages, promoting M1 polarization through STAT1 succinylation and enhanced transcription of glycolytic genes, thereby exacerbating pulmonary inflammation.^77^ These findings elucidate how epigenetic regulation integrates environmental signals to modulate immune cell function and phenotype in SI-ALI, as summarized in Table 1.

**Table 1:** Epigenetic regulatory mechanisms in SI-ALI

Epigenetic mechanism	Key regulators	Target genes/Pathways	Cell types	Biological effects
DNA methylation	DNMT1	TLR2, SOCS3 promoters	PBMCs, macrophages	pro-inflammatory gene overexpression, immune homeostasis disruption
Histone methylation	EZH2	USP10, GPX4	alveolar epithelial cells	ferroptosis induction, impaired antioxidant defense
Histone acetylation	HDAC3	ATG5, H3K27ac	macrophages	enhanced autophagy, pyroptosis inhibition, reduced oxidative stress
Histone lactylation	KAT2B, RBM25-ACLY axis	EGR1, H3K18la	endothelial cells	glycocalyx degradation, increased vascular permeability
Histone demethylation	KDM6B	MFN1, H3K27me3	macrophages	promoted mitophagy, apoptosis inhibition
MicroRNA regulation	miR-146a, miR-150, miR-223	NF-κB, c-Myb, neutrophil function	immune cells	inflammation regulation, immune response remodeling
lncRNA regulation	HOTAIRM1, NEAT1	inflammatory signaling pathways	immune cells, endothelial cells	amplified inflammation, vascular barrier disruption
circRNA regulation	circEXOC5, circMAPK1	Nrf2, KDM2B/WNK1	endothelial cells	pyroptosis activation, reduced antioxidant capacity
RNA methylation	m6A modification enzyme complex	inflammation-related mRNAs	immune cells	pro-inflammatory response, organ dysfunction

## Epigenetic biomarkers in risk stratification and prognostic prediction

### DNA methylation biomarkers

Epigenetic regulation, particularly DNA methylation, has emerged as a critical mechanism influencing gene expression without altering the DNA sequence, thereby linking genetic predisposition with environmental stimuli in complex diseases such as SI-ALI. Recent advances in epigenome-wide association studies (EWASs) have enabled comprehensive profiling of genome-wide DNA methylation patterns in affected tissues and circulating immune cells. In patients with SI-ALI, EWAS performed on peripheral blood mononuclear cells (PBMCs) and lung tissue samples has revealed distinct differentially methylated regions (DMRs) associated with disease presence and progression. These DMRs represent loci exhibiting significant methylation differences between patients with SI-ALI and healthy controls, or across severity strata within patient cohorts. Identification of such DMRs is crucial, as they are frequently located within regulatory regions of genes involved in immune responses, inflammation, and tissue repair—processes central to SI-ALI pathophysiology. For instance, methylation alterations in promoter or enhancer regions can modulate transcriptional activity of key genes, thereby influencing the host inflammatory milieu and susceptibility to organ injury. Consequently, EWAS applications in SI-ALI provide high-resolution maps of epigenetic alterations that may serve as biomarkers for early detection, risk stratification, and therapeutic targeting. Furthermore, these findings underscore the dynamic nature of the epigenome in response to septic insult and highlight the potential of DNA methylation analysis as a non-invasive tool for monitoring disease trajectory and treatment response.^74^

Among differentially methylated loci identified through genome-wide methylation screening, TLR2 and SOCS3 have garnered particular attention due to their central roles in balancing host defense and inflammation. Studies have demonstrated that methylation levels in the promoter regions of these genes are significantly correlated with disease severity, organ failure scores, and 28-day mortality in patients with SI-ALI, offering novel molecular dimensions for early risk stratification. TLR2, as a pattern recognition receptor, recognizes pathogen components and initiates innate immune responses; aberrant methylation of its promoter region can lead to gene expression dysregulation, thereby amplifying or dampening inflammatory reactions. SOCS3, conversely, functions as a negative feedback regulator of cytokine signaling, controlling the intensity and duration of inflammatory responses; hypermethylation of its promoter is often accompanied by gene silencing, consequently triggering uncontrolled inflammation and tissue damage. Clinical data further confirm that TLR2 and SOCS3 promoter methylation status aligns closely with traditional prognostic indicators such as the Sequential Organ Failure Assessment (SOFA) score and independently predicts 28-day mortality in patients with SI-ALI. This association suggests that epigenetic modifications at these loci are not random events but rather molecular snapshots of underlying pathophysiological states, potentially serving as biomarkers for early prognostic assessment. Compared to conventional clinical scoring systems, DNA methylation markers offer a novel molecular-level evaluation dimension, facilitating more precise and timely therapeutic interventions. Moreover, given the dynamic and reversible nature of DNA methylation, targeting these epigenetic marks may also represent emerging therapeutic strategies for improving SI-ALI outcomes.^74^

Building upon the identification of individual DMRs associated with SI-ALI severity and outcomes, researchers have developed composite methylation risk score models that integrate multiple epigenetic markers to enhance prognostic accuracy.^78^ These models aggregate methylation data from several key DMRs, capturing a broader spectrum of epigenetic alterations that collectively reflect the complex biological processes driving disease progression. In independent validation cohorts, methylation risk scores have consistently outperformed traditional clinical indicators—such as SOFA or APACHE II scores—in predicting patient outcomes, including mortality and organ failure. This superior predictive performance is attributed to the molecular specificity and sensitivity of DNA methylation patterns, which can detect subtle changes in gene regulation before overt clinical deterioration manifests. Additionally, methylation risk scores offer the advantage of being measurable from peripheral blood samples, facilitating minimally invasive monitoring. Integration of these epigenetic biomarkers into clinical practice could revolutionize risk stratification by enabling personalized prognostic assessment and guiding targeted interventions. Furthermore, these models provide a framework for identifying patient subsets that may benefit from epigenetic therapies or other precision medicine approaches. However, further large-scale prospective studies are needed to standardize methylation detection methods, validate risk score thresholds, and assess their utility across diverse patient populations.

### Circulating non-coding RNA biomarkers

Circulating microRNAs have emerged as key epigenetic biomarkers in SI-ALI, reflecting the complex immunopathological states underlying this syndrome. Among these, miR-146a, miR-150, and miR-223 consistently exhibit significant expression alterations in plasma from patients with sepsis and ALI, demonstrating correlations with distinct inflammatory phenotypes and clinical outcomes. For instance, miR-146a is known to modulate innate immune responses by targeting key signaling molecules in the NF-κB pathway, thereby influencing the inflammatory milieu characteristic of sepsis and ALI. Elevated plasma levels of miR-146a have been associated with regulation of the cytokine storm and immune dysregulation, serving as a potential indicator of inflammatory endotypes and lung injury severity.^41^ Similarly, miR-150—which plays roles in immune cell differentiation and function—exhibits reduced plasma expression in severe sepsis and ALI cases, with its downregulation correlated with poorer prognosis and increased mortality. This suggests that miR-150 may serve as a biomarker for immunosuppression and disease progression.^79^ miR-223, primarily expressed in neutrophils, participates in regulating neutrophil activation and function—processes critical to ALI pathogenesis. Alterations in its circulating levels reflect neutrophil-mediated inflammatory responses and correlate with the extent of lung injury. The plasma expression profiles of these miRNAs not only reflect underlying immune dysregulation but also provide stratification tools for risk assessment and prognostic evaluation in SI-ALI. Their stability in circulation and specificity to immune pathways highlight their potential utility as non-invasive biomarkers in precision medicine for sepsis and ALI, facilitating early diagnosis, disease progression monitoring, and therapeutic targeting.^80^

The prognostic significance of miR-150 in sepsis and ALI lies in its inverse correlation with patient survival, wherein reduced circulating levels portend increased mortality. Mechanistically, miR-150 exerts immunomodulatory effects by targeting transcription factors such as c-Myb, which is essential for hematopoietic cell proliferation and differentiation. Downregulation of miR-150 may lead to dysregulated immune cell development and impaired host defense, exacerbating the immunosuppressive phase of sepsis and contributing to adverse outcomes. Clinical studies indicate that patients with lower plasma miR-150 levels exhibit more pronounced systemic inflammation and impaired immune recovery, correlating with increased risk of multi-organ failure and mortality. Concurrently, miR-223—a myeloid-specific miRNA—orchestrates neutrophil function by modulating granulopoiesis and inflammatory responses. It regulates neutrophil activation, chemotaxis, and apoptosis—processes central to ALI pathophysiology. Elevated or dysregulated circulating miR-223 levels reflect aberrant neutrophil activity, potentially contributing to excessive pulmonary inflammation and tissue damage. Experimental models demonstrate that miR-223 deficiency exacerbates lung injury through promoting neutrophil infiltration and inflammatory cytokine release, underscoring its protective role. Therefore, dynamic expression of miR-150 and miR-223 in plasma not only serves as biomarkers of disease severity but also provides insights into the immune mechanisms driving sepsis-induced lung injury. Targeting these miRNAs may offer therapeutic avenues for modulating immune responses and improving patient survival.^81^^,^^82^

LncRNAs, including HOTAIRM1 and NEAT1, have emerged as novel circulating biomarkers whose expression changes correlate with systemic inflammatory intensity and pulmonary vascular permeability alterations in patients with SI-ALI.^83^ Beyond miRNAs, circulating lncRNAs have gained recognition as epigenetic biomarkers in SI-ALI, reflecting systemic inflammation and vascular dysfunction. LncRNAs such as HOTAIRM1 and NEAT1 exhibit altered plasma expression profiles in patients with SI-ALI, correlating with the severity of systemic inflammatory responses and disruption of the pulmonary endothelial barrier. HOTAIRM1, known for its role in myeloid cell differentiation and inflammatory regulation, is upregulated in plasma from patients with ALI, paralleling elevated inflammatory cytokine levels and increased endothelial permeability. This suggests that HOTAIRM1 may contribute to the dysregulated immune responses and vascular leakage characteristic of ALI.^84^ NEAT1, a nuclear-enriched transcript involved in paraspeckle formation and gene expression regulation, similarly exhibits elevated circulating levels during sepsis and ALI. Its expression correlates with markers of endothelial dysfunction and lung injury severity, implicating NEAT1 in the regulation of pulmonary vascular integrity and inflammatory signaling pathways.^85^ The stability of these lncRNAs in biological fluids and their association with key pathological features of SI-ALI position them as promising biomarkers for risk stratification and therapeutic monitoring. Furthermore, their mechanistic involvement in immune and endothelial cell regulation presents potential targets for precision interventions aimed at mitigating lung injury and improving patient outcomes.

### Histone modification-related biomarkers

Histone modifications represent a critical epigenetic mechanism that influences gene expression without altering the underlying DNA sequence, primarily through post-translational modifications of histone tails—including methylation, acetylation, and lactylation. In the context of SI-ALI, these modifications have emerged as key regulators of inflammatory responses, cell death pathways, and immune cell function, thereby offering promising avenues for risk stratification and targeted therapy.^86^ Although direct detection of histone modifications in circulating blood remains technically challenging due to their intracellular and dynamic nature, surrogate biomarkers can be assessed by measuring the activity or expression levels of histone-modifying enzymes, including HDACs and histone methyltransferases. For instance, EZH—the histone methyltransferase catalyzing H3K27me3—is upregulated in lung tissue during SI-ALI and promotes ferroptosis in alveolar epithelial cells by suppressing USP10 expression, leading to destabilization of the antioxidant enzyme GPX4 and exacerbating oxidative damage.^66^ This mechanistic insight underscores the potential of EZH2 and its associated histone methylation marks as biomarkers reflecting disease severity and as therapeutic targets for mitigating epithelial cell death.

Similarly, inhibition of HDAC3 with the small molecule BRD3308 has been shown to attenuate lung injury in sepsis models by suppressing NLRP3 inflammasome-mediated pyroptosis in macrophages. This effect is mediated through increased H3K27ac, which upregulates autophagy-related gene-5, enhancing autophagic flux and restoring redox balance in immune cells.^65^ These findings highlight the dual role of histone acetylation in regulating inflammatory cell death and oxidative stress, suggesting that HDAC activity levels may serve as surrogate biomarkers of inflammatory status and responsiveness to epigenetic therapies in SI-ALI. Furthermore, KDM6B—a histone demethylase responsible for removing the repressive H3K27me3 mark—has been implicated in regulating macrophage apoptosis and mitophagy during sepsis-induced ALI. KDM6B expression is elevated in septic lung tissue, and its knockdown increases H3K27me3 enrichment at the MFN1 promoter, suppressing MFN1 transcription, reducing macrophage apoptosis, and promoting mitophagy—thereby alleviating lung injury and improving survival in septic mice.^87^ This epigenetic regulation of macrophage fate determination through dynamic histone methylation provides a potential biomarker axis for identifying patients with dysregulated immune responses and for guiding therapies aimed at restoring macrophage homeostasis.

In PBMCs, histone H3 lysine 18 lactylation (H3K18la) has been identified as a novel epigenetic modification associated with sepsis severity. Elevated H3K18la levels positively correlate with organ failure scores and inflammatory cytokine profiles, while the ratio of H3K18la to acetylation may serve as an independent biomarker for diagnosing sepsis and septic shock.^88^ This lactylation mark reflects metabolic reprogramming in immune cells and links cellular metabolism to epigenetic regulation of genes involved in macrophage polarization and immunosuppression. Consequently, the dynamic interplay between histone lactylation and acetylation represents a promising biomarker axis for risk stratification and may inform the timing and selection of immunomodulatory therapies. During sepsis-induced lung injury, histone modifications also intersect with metabolic pathways in endothelial cells. For instance, lactate accumulation promotes histone H3K18 lactylation at the EGR1 promoter, enhancing its transcription and subsequently upregulating heparanase—which degrades the pulmonary endothelial glycocalyx, exacerbating vascular permeability and lung injury.^72^ KAT2B has been identified as the lactyltransferase mediating this modification, further expanding the repertoire of histone-modifying enzymes that could serve as biomarkers or therapeutic targets in SI-ALI.

**Figure 3:**
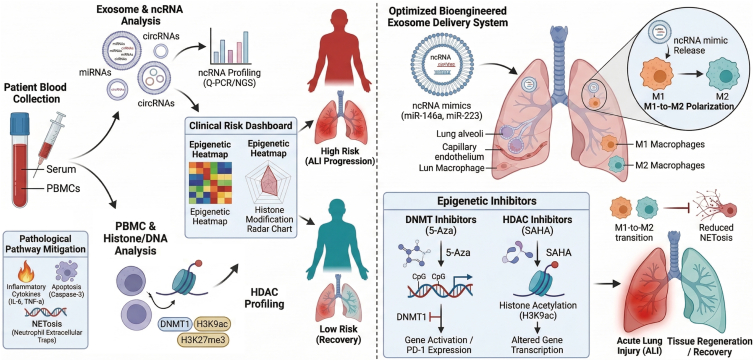
Epigenetic biomarker-guided precision diagnosis and therapeutic strategies for SI-ALI This figure depicts a precision medicine workflow for SI-ALI. Patient blood is collected for ncRNA profiling (miRNAs, circRNAs) via Q-PCR/NGS, and PBMCs are analyzed for histone/DNA modification markers. Epigenetic data stratify patients into high-risk and low-risk categories via a clinical dashboard integrating epidemic heatmaps and radar charts. DNMT inhibitors (5-Aza) and HDAC inhibitors (SAHA) modulate DNMT1, H3K9ac, H3K27me3, and CpG, leading to altered gene transcription. Therapeutic outcomes include M1-to-M2 macrophage polarization, reduced NETosis, suppression of inflammatory cytokines (IL-6, TNF-α), apoptosis modulation (Caspase-3), PD-1 expression changes, and acute lung tissue regeneration/recovery. Exosome delivery systems and bioengineered ncRNA/miRNA/circRNA mimics serve as optimized therapeutic platforms.

While direct measurement of circulating histone modifications remains limited, assessing the expression and enzymatic activity of histone-modifying enzymes—such as HDACs and methyltransferases—in peripheral immune cells offers a feasible alternative. Notably, reduced HDAC2 activity in PBMCs has been associated with glucocorticoid (GC) resistance in sepsis, thereby identifying a high-risk patient subset unlikely to respond to conventional anti-inflammatory therapy.^74^ This suggests that HDAC2 activity may serve as a predictive biomarker of therapeutic responsiveness, enabling personalized treatment strategies. Beyond enzyme activity, epigenetic inhibitors targeting histone modifications have demonstrated therapeutic potential in preclinical models of SI-ALI. HDAC inhibitors and methyltransferase inhibitors can modulate inflammatory gene expression, reduce oxidative stress, and prevent cell death, thereby attenuating lung injury and improving survival.^66^ Table 2 provides a systematic summary of these epigenetic biomarkers for risk stratification and identification of patients who may benefit from epigenetic therapies. The clinical application workflow integrating these biomarkers into precision medicine strategies is illustrated in Figure 3.

**Table 2:** Epigenetic biomarkers in SI-ALI and their clinical applications

Biomarker type	Specific biomarker	Sample source	Clinical relevance	Predictive value	Detection method
DNA Methylation	TLR2/SOCS3 methylation levels	PBMCs	positively correlated with SOFA score, disease severity	independent predictor of 28-day mortality	pyrosequencing
DNA methylation	multi-DMRs composite risk score	whole blood/PBMCs	integrates multi-gene methylation information	superior prognostic performance vs. traditional clinical scores	EWAS, methylation array
miRNA	miR-146a downregulation	plasma	reflects hyperinflammatory endotype, immune dysregulation	marker of disease severity	qRT-PCR
miRNA	miR-150 downregulation	plasma	associated with immunosuppression	increased 28-day mortality	qRT-PCR
miRNA	miR-223 upregulation	plasma	reflects neutrophil activation status	correlated with lung injury severity	qRT-PCR
lncRNA	HOTAIRM1, NEAT1 upregulation	plasma	associated with systemic inflammation, endothelial dysfunction	marker of lung injury severity	qRT-PCR
Histone modification	H3K18la levels	PBMCs	positively correlated with SOFA score, organ failure	diagnostic marker for septic shock	ChIP-qPCR (PBMC lysates)
Histone-modifying enzyme	HDAC2 activity reduction	PBMCs	associated with glucocorticoid resistance	predicts non-response to corticosteroid therapy	enzyme activity assay
Histone-modifying enzyme	EZH2 upregulation	lung tissue/BALF cells	promotes alveolar epithelial cell ferroptosis	correlated with lung injury severity	IHC, western blot, qRT-PCR

## Epigenetic biomarker-guided targeted therapeutic strategies

### Therapeutic potential of epigenetic drugs in sepsis-induced acute lung injury

Among epigenetic drugs, DNMTis such as 5-azacytidine and HDACis including vorinostat and trichostatin A have demonstrated significant efficacy in preclinical models of ALI by attenuating inflammation and alleviating lung tissue damage. These agents primarily function by reversing pathological epigenetic silencing or inhibiting aberrant activation of inflammatory gene transcription, thereby restoring immune homeostasis and mitigating the deleterious inflammatory cascades characteristic of SI-ALI.

DNA methylation, catalyzed by DNMTs, typically suppresses gene expression through addition of methyl groups to cytosine residues within CpG islands. In sepsis and ALI, hypermethylation of anti-inflammatory genes or hypomethylation of pro-inflammatory genes may exacerbate lung injury. For instance, vitexin—a natural compound identified through network pharmacology—has been shown to modulate the SNHG1/DNMT1/miR-495 axis, reducing inflammation and pyroptosis in sepsis-associated ALI through effects on DNA methylation patterns.^64^ This highlights the potential of DNMT inhibitors to restore balanced gene expression profiles in pulmonary tissues during sepsis.

HDAC inhibitors such as vorinostat and trichostatin A exert their effects by increasing histone acetylation levels, which generally promotes transcriptional activation of genes involved in anti-inflammatory responses and cellular repair. A notable example is BRD3308, a selective HDAC3 inhibitor, which has been demonstrated to attenuate lung tissue damage and inflammatory infiltration in models of sepsis-induced ALI. Mechanistically, BRD3308 enhances H3K27ac, leading to upregulation of autophagy-related genes such as ATG5. This activation of autophagy reduces oxidative stress and pyroptosis in macrophages—key contributors to septic lung injury.^65^ These findings underscore the capacity of HDACis to modulate epigenetic marks that govern immune cell function and inflammatory responses within the pulmonary microenvironment. The therapeutic efficacy of these epigenetic drugs is further supported by their ability to reshape immune cell phenotypes. For instance, HDAC inhibition can promote macrophage polarization toward an anti-inflammatory M2 phenotype, thereby restraining the excessive inflammatory responses that drive tissue damage in SI-ALI. Similarly, DNMT inhibition can restore expression of microRNAs and lncRNAs that regulate immune signaling pathways, as observed with vitexin modulation of the SNHG1/miR-495 axis.^64^ Thus, these epigenetic interventions operate at multiple regulatory levels to recalibrate immune homeostasis.

Looking forward, integration of patient-specific epigenetic profiles holds promise for precision medicine approaches in SI-ALI. Epigenetic biomarkers—such as gene-specific DNA methylation patterns or histone modification signatures—could guide selection of appropriate epigenetic drugs based on an individual patient’s molecular profile. For example, patients exhibiting hypermethylation of protective genes might benefit from DNMT inhibitors, while those with dysregulated histone acetylation could be candidates for HDAC inhibition. Such “biomarker-drug” matching strategies could optimize therapeutic efficacy while minimizing off-target effects.^24^ Furthermore, the interplay between metabolic reprogramming and epigenetic modifications in SI-ALI suggests additional therapeutic avenues. Recent studies have identified histone lactylation as a novel epigenetic mark linking metabolic changes to gene expression in sepsis. Lactate accumulation in pulmonary endothelial cells promotes H3K18la, enhancing transcription of genes such as EGR1 that drive endothelial glycocalyx degradation and lung injury.^72^ Targeting enzymes involved in lactylation or associated metabolic pathways may complement classical epigenetic drugs, enabling more comprehensive modulation of the epigenetic landscape in SI-ALI.

### Therapeutic applications of miRNA mimics and antagonists

Therapeutic strategies utilizing miRNA mimics and antagonists offer promising avenues for risk stratification and targeted intervention in ALI. For protective miRNAs that are downregulated during SI-ALI—such as miR-146a—administration of synthetic miRNA mimics can restore their anti-inflammatory feedback loops, thereby attenuating excessive inflammation and tissue damage. Conversely, for pathogenic miRNAs that are upregulated and exacerbate lung injury—including certain pro-inflammatory miRNAs such as miR-1-3p and miR-27a-5p—inhibition using antagomiRs can block their deleterious effects. For instance, miR-21-5p—downregulated in alveolar epithelial cells during lipopolysaccharide-induced injury—has been shown to exert protective effects by targeting SLC16A10, thereby reducing pro-inflammatory cytokine production and cellular damage. Upregulation of miR-21-5p via mimics decreased IL-1β and TNF-α expression, highlighting its therapeutic potential in mitigating epithelial inflammation.^89^ Similarly, miR-130b-3p mimics engineered for enhanced stability effectively suppressed eCIRP-mediated inflammation and ALI in murine sepsis models, demonstrating the feasibility of miRNA mimic-based therapeutics.^90^ Conversely, miR-1-3p—elevated in plasma exosomes during sepsis—promotes endothelial dysfunction by targeting SERP1, leading to increased apoptosis, cytoskeletal contraction, and elevated barrier permeability. Accordingly, antagonizing miR-1-3p restored endothelial integrity and reduced vascular leakage in ALI.^91^ Likewise, miR-27a-5p is upregulated in pulmonary microvascular endothelial cells during sepsis and promotes increased leukocyte adhesion and infiltration; inhibition of miR-27a-5p attenuated these pathological processes, suggesting that antagonists targeting miR-27a-5p may alleviate endothelial dysfunction in ALI.^92^

A key challenge in miRNA-based therapeutics lies in achieving cell-type specificity to maximize efficacy while minimizing systemic side effects. Targeted delivery systems, such as macrophage membrane biomimetic nanoparticles (MM NPs), have been developed to deliver miRNA mimics or inhibitors directly to pulmonary macrophages or neutrophils, thereby modulating inflammatory signaling pathways locally within the lung microenvironment. For example, MM NPs loaded with miR-125a-5p inhibitors or miR-221-3p mimics attenuated LPS-induced ALI by modulating macrophage and neutrophil responses, underscoring the potential of cell-specific delivery platforms for precision therapeutics.^93^

Exosome-based delivery systems derived from mesenchymal stem cells (MSCs) or adipose-derived stem cells (ADSCs) also offer promising vehicles for miRNA therapeutics. MSC-derived exosomes carrying miR-125b-5p have been shown to inhibit macrophage pyroptosis through STAT3 downregulation, thereby attenuating sepsis-induced ALI.^94^ Similarly, ADSC-derived exosomes enriched with miR-125b-5p alleviated ferroptosis in pulmonary microvascular endothelial cells via the Keap1/Nrf2/GPX4 pathway, reducing oxidative stress and inflammation in septic lung injury.^95^ These findings highlight the dual role of exosomal miRNAs as both biomarkers and therapeutic agents. Furthermore, modulation of miRNAs involved in endothelial-to-mesenchymal transition and barrier integrity—such as miR-23b-3p targeting SMAD3—has demonstrated efficacy in restoring endothelial homeostasis and reducing vascular leakage in sepsis-induced ALI.^96^ Regulating such miRNAs using agomiRs or antagomiRs may fine-tune the balance between injury and repair mechanisms within the lung.^48^ Vitamin D supplementation has also been shown to exert protective effects in ALI through enhancing miR-149-5p expression, which suppresses endoplasmic reticulum stress pathways, further illustrating the therapeutic potential of integrating miRNA modulation with other interventions.^97^

### Epigenetic biomarkers for predicting response to existing therapies

GCs remain a cornerstone of immunomodulatory therapy for SI-ALI, aimed at attenuating the excessive inflammatory responses characteristic of this condition. However, clinical outcomes following GC treatment exhibit substantial heterogeneity, with some patients demonstrating significant improvement while others derive minimal or no benefit. This variability underscores the pressing need for predictive biomarkers capable of stratifying patients likely to respond to GC therapy, thereby optimizing therapeutic efficacy while minimizing unnecessary exposure to potential side effects. Epigenetic modifications—particularly DNA methylation patterns and microRNA expression profiles—have emerged as promising candidates for such predictive biomarkers. For instance, methylation alterations in the FK506 binding protein 5 (FKBP5) gene, a known regulator of GC receptor sensitivity, have been associated with variations in GC responsiveness across various inflammatory conditions. Hypomethylation of the FKBP5 promoter region may enhance gene expression, potentially modulating the GC receptor complex and influencing steroid sensitivity.^98^ Similarly, specific miRNAs have been implicated in regulating inflammatory pathways and GC receptor signaling. Distinct miRNA signatures—such as altered levels of miR-146a and miR-155—correlate with differential inflammatory responses and may serve as indicators of GC therapeutic efficacy.^99^ These epigenetic marks not only reflect the dynamic interplay between the host immune system and therapeutic agents but also provide mechanistic insights into the molecular basis of treatment resistance or sensitivity. Integration of epigenetic profiling into clinical practice could enable personalized GC therapy in SI-ALI, tailoring immunomodulatory regimens according to patients’ epigenetic landscapes and thereby improving outcomes.

Beyond GCs, β-adrenergic agonists represent another therapeutic avenue in SI-ALI, primarily functioning through enhancement of alveolar fluid clearance and improvement of pulmonary edema resolution. However, similar to steroids, patient responses to β-agonists vary considerably, and indiscriminate use may lead to suboptimal efficacy or adverse effects.^100^ Epigenetic biomarkers offer a promising strategy for predicting which patients will benefit from β-agonist therapy. DNA methylation and histone modification patterns within genes involved in β-adrenergic signaling pathways—such as ADRB2—may influence receptor expression and downstream signaling efficiency. For instance, hypermethylation of the ADRB2 promoter could reduce receptor availability, thereby diminishing β-agonist therapeutic efficacy.^101^ Additionally, miRNAs targeting components of the β-adrenergic pathway may modulate receptor sensitivity and signal transduction.^102^ Analysis of these epigenetic features could stratify patients into responders and non-responders, enabling precision medicine approaches that maximize therapeutic benefit while minimizing unnecessary drug exposure. Furthermore, epigenetic markers may extend to other targeted therapies under investigation in SI-ALI, including agents modulating inflammatory cascades or endothelial barrier function. By integrating epigenetic data with clinical parameters, clinicians could develop robust predictive models to guide treatment selection, thereby avoiding ineffective therapies and reducing healthcare costs. This approach aligns with the broader paradigm of pharmaco-epigenetics, which has demonstrated promise in predicting drug response and resistance in other diseases such as cancer, autoimmune disorders, and metabolic conditions. Although research specifically addressing epigenetic predictors in SI-ALI remains limited, emerging evidence from related fields supports the feasibility and potential clinical utility of this strategy. Future studies should focus on validating candidate epigenetic biomarkers in well-characterized SI-ALI cohorts and exploring their mechanistic roles in modulating drug responses, with the key therapeutic strategies summarized in Table 3.

**Table 3:** Epigenetic-targeted therapeutic strategies in SI-ALI

Therapeutic strategy	Drug/Intervention	Target	Mechanism of action	Cellular target	Biological effects
DNMT inhibitor	5-azacytidine, Vitexin	DNMT1, SNHG1/DNMT1/miR-495 axis	reverses aberrant DNA methylation patterns	alveolar epithelial cells	attenuates inflammation, inhibits pyroptosis
HDAC inhibitor	BRD3308	HDAC3	increases H3K27ac, upregulates ATG5 expression	macrophages	enhances autophagy, reduces oxidative stress, inhibits pyroptosis
EZH2 inhibitor	DZNep	EZH2	reduces H3K27me3, upregulates GPX4 expression	alveolar epithelial cells	inhibits ferroptosis, restores antioxidant capacity
EZH2 inhibitor	Unnamed EZH2 inhibitor	EZH2	promotes macrophage M2 polarization	macrophages	attenuates inflammation, inhibits pulmonary fibrosis
miRNA mimic	miR-146a mimic	NF-κB pathway	restores negative feedback regulation	macrophages	suppresses excessive inflammatory response
miRNA mimic	miR-125b-5p mimic	STAT3	inhibits macrophage pyroptosis	macrophages	attenuates lung injury
miRNA antagonist	anti-miR-1-3p	SERP1	restores endothelial cell function	endothelial cells	reduces apoptosis, maintains barrier integrity
Targeted delivery system	Macrophage biomimetic nanoparticles	pulmonary macrophages	delivers miRNA modulators to target cells	pulmonary macrophages	achieves localized immunomodulation
Stem cell exosomes	MSC-exo miR-125b-5p	STAT3	inhibits macrophage pyroptosis	macrophages	attenuates lung injury
Stem cell exosomes	ADSC-exo miR-125b-5p	Keap1/Nrf2/GPX4 axis	inhibits endothelial cell ferroptosis	endothelial cells	reduces oxidative stress

## Detection technologies and translational challenges for epigenetic biomarkers

### Detection platforms and analytical methods

High-throughput technologies have revolutionized the discovery of epigenetic biomarkers in SI-ALI by enabling comprehensive, unbiased analysis of genome-wide epigenetic modifications and gene expression changes. These platforms include methylation arrays, whole-genome bisulfite sequencing (WGBS), chromatin immunoprecipitation sequencing (ChIP-seq), and RNA sequencing (RNA-seq), which collectively facilitate unbiased biomarker discovery. Methylation arrays and WGBS enable detailed mapping of DNA methylation patterns at single-base resolution, which is essential for identifying DMRs associated with disease states. For instance, WGBS can reveal global and site-specific methylation alterations in lung tissue or circulating immune cells during sepsis, providing insights into the epigenetic reprogramming underlying immune dysregulation and tissue injury.^103^ ChIP-seq complements methylation analysis by profiling histone modifications and transcription factor binding, which is crucial for understanding chromatin state changes that regulate gene expression in SI-ALI.^104^ RNA-seq further integrates these epigenetic data by quantifying transcriptomic alterations—including coding and non-coding RNAs such as microRNAs that have emerged as key epigenetic regulators and potential biomarkers.^105^ Integration of these high-throughput platforms facilitates identification of novel epigenetic signatures reflecting the complex interplay between metabolic shifts, immune responses, and pulmonary tissue remodeling in sepsis. For example, whole-transcriptome analysis has identified lactylation-related genes such as RBM25 and ACLY, whose expression correlates with sepsis prognosis and ALI severity, highlighting metabolic-epigenetic axes as promising targets for biomarker development and therapeutic intervention.^73^ Similarly, epigenetic profiling of histone lactylation at specific residues such as H3K18 has elucidated mechanisms by which metabolic byproducts such as lactate exacerbate pulmonary microvascular endothelial dysfunction and glycocalyx degradation during sepsis.^72^ These findings underscore the power of high-throughput epigenomic and transcriptomic technologies in revealing molecular pathways and biomarkers inaccessible through conventional approaches, thereby advancing precision medicine strategies for SI-ALI.

While high-throughput platforms are indispensable for biomarker discovery, translation of epigenetic biomarkers into clinical application requires rapid, sensitive, and cost-effective assays suitable for point-of-care or routine laboratory use—such as development of qPCR-based, pyrosequencing-based, or digital PCR-based assays for validated biomarkers. Quantitative PCR assays targeting specific miRNAs have become cornerstone methods for rapid biomarker detection due to their high sensitivity, specificity, and relatively low cost. miRNAs exhibit stability in biofluids and reflect dynamic changes in immune and inflammatory pathways during sepsis and SI-ALI, positioning them as ideal candidates for clinical monitoring. For DNA methylation biomarkers, pyrosequencing provides a robust method for quantifying methylation levels at specific CpG sites with high accuracy and throughput, enabling targeted validation of candidate epigenetic marks identified through genome-wide studies. Digital PCR further enhances detection sensitivity and quantitative precision, particularly for low-abundance targets or rare epigenetic variants, which is critical for early diagnosis and risk stratification in septic patients. Development of these rapid assays facilitates timely decision-making in intensive care settings, enabling clinicians to stratify patients according to epigenetic risk profiles and adjust interventions accordingly. For instance, lactylation-based gene signatures involving RBM25 and ACLY have been validated through qRT-PCR in murine models of sepsis-induced ALI, demonstrating the feasibility of translating complex epigenetic discoveries into practical diagnostic tools.^73^ Furthermore, therapeutic effects of interventions targeting lactate production or epigenetic regulators such as KAT2B—identified through mechanistic studies—can be monitored in real-time using these rapid assays.^72^ Thus, rapid detection technologies bridge the gap between discovery and clinical application, enabling precision medicine approaches to improve outcomes in SI-ALI.

Single-cell epigenomic technologies have emerged as transformative tools for dissecting the cellular heterogeneity and complex immune landscape characteristic of SI-ALI. Unlike bulk analyses, single-cell approaches enable resolution of epigenetic and transcriptomic profiles at the individual cell level, revealing distinct immune cell subsets and their unique regulatory states within the inflammatory pulmonary microenvironment. This is particularly critical in SI-ALI, where regionalized immune responses and diverse cell types—including alveolar macrophages, neutrophils, endothelial cells, and various lymphocyte subsets—differentially contribute to disease progression and resolution. Single-cell bisulfite sequencing (scBS-seq), single-cell ATAC sequencing (scATAC-seq), and single-cell RNA sequencing (scRNA-seq) integrated with epigenetic profiling enable identification of cell type-specific DNA methylation patterns, chromatin accessibility landscapes, and gene expression signatures. For instance, single-cell analyses can reveal how metabolic-epigenetic circuits—such as the RBM25-ACLY axis and histone lactylation modifications—are differentially regulated across immune cell subsets, influencing their functional contributions to lung injury.^106^ Furthermore, single-cell epigenomics facilitates discovery of novel biomarkers reflecting immune response heterogeneity, enabling more precise risk stratification and targeted therapeutics. By capturing dynamic interactions between immune cells and the pulmonary microenvironment at high resolution, single-cell approaches hold promise for identifying epigenetic biomarkers predictive of delayed ARDS onset and survival outcomes, as demonstrated by multi-omics studies involving genes such as WNT9A in ARDS pathogenesis.^107^ Consequently, single-cell epigenomic technologies provide a powerful platform for advancing precision medicine in SI-ALI by enabling development of refined, cell type-specific biomarkers and therapeutic targets that address the complexity of sepsis-induced lung injury.

### Major challenges in translation

#### Sample source and standardization: Discrepancies in epigenetic information between blood (whole blood, PBMCs) and lung tissue samples; need for standardized sampling timing and processing methods to ensure comparability of results

One of the foremost challenges in translating epigenetic biomarkers for risk stratification and targeted therapy in SI-ALI lies in the heterogeneity of sample sources and the lack of standardized protocols. Epigenetic modifications—including DNA methylation, histone modifications, and RNA methylation—exhibit high tissue and cell type specificity, reflecting local microenvironments and cellular contexts. In SI-ALI, the lung tissue itself undergoes complex immunological and structural changes, while peripheral blood samples such as whole blood or PBMCs may capture systemic immune alterations but do not fully represent pulmonary-specific epigenetic states. For instance, studies have demonstrated that EZH2—an epigenetic regulator mediating histone methylation—is upregulated in lung tissue during SI-ALI and modulates alveolar epithelial cell ferroptosis and macrophage polarization, processes critical to lung injury pathogenesis.^66^ Conversely, peripheral blood samples may reflect the systemic inflammatory and immunosuppressive phases of sepsis, with epigenetic alterations in circulating immune cells influencing disease progression.^74^ This discordance between pulmonary tissue and blood epigenetic profiles complicates biomarker development, as peripheral samples are more clinically accessible yet may fail to capture organ-specific pathophysiological changes.

Furthermore, the timing of sample collection is critical due to the dynamic nature of epigenetic modifications over the course of sepsis progression. The early phase of sepsis is characterized by hyperinflammation, whereas later stages involve immunosuppression and tissue repair, each associated with distinct epigenetic landscapes. Without standardized sampling timing, comparisons across different studies and patient cohorts become unreliable. Additionally, pre-analytical variables such as sample processing, storage conditions, and handling methods—for instance, separation of PBMCs versus whole blood analysis—can introduce variability in epigenetic readouts. For example, histone modification levels and RNA methylation patterns may be sensitive to freeze-thaw cycles and processing delays, potentially biasing results.^71^ Therefore, establishment of rigorous, standardized protocols for sample collection, processing, and storage is essential to ensure reproducibility and comparability of epigenetic biomarker data across different clinical settings.

Moreover, cellular heterogeneity within lung tissue samples presents additional challenges. Lung tissue comprises multiple cell types—including alveolar epithelial cells, endothelial cells, macrophages, and fibroblasts—each possessing distinct epigenetic signatures and playing different roles in ALI pathogenesis.^77^ Bulk analysis of tissues may mask cell-specific epigenetic alterations critical for understanding disease mechanisms and identifying precise therapeutic targets. Single-cell epigenomic technologies offer promise for addressing this issue but are currently limited by cost, technical complexity, and the requirement for fresh tissue samples, which are difficult to obtain from critically ill patients. In contrast, blood samples allow for repeated, minimally invasive sampling but require careful interpretation to link systemic epigenetic changes to pulmonary pathology.

#### Dynamic changes and temporal specificity: Rapid evolution of epigenetic marks during disease progression; need to define their dynamic trajectories to determine optimal detection windows

Epigenetic modifications in SI-ALI are not static but rather exhibit rapid and dynamic changes throughout the disease course, reflecting the host’s evolving response to infection and tissue injury. This temporal variability poses a significant challenge for the clinical application of epigenetic biomarkers in risk stratification and targeted therapy. Understanding the kinetics and trajectories of epigenetic alterations is essential for determining optimal time windows for biomarker detection and therapeutic intervention. Sepsis triggers an initial hyperinflammatory phase characterized by activation of pro-inflammatory genes, which are often regulated by epigenetic mechanisms such as histone acetylation and methylation. For instance, histone lactylation—a novel epigenetic modification induced by elevated lactate levels during sepsis—has been shown to promote transcriptional reprogramming in pulmonary endothelial cells, exacerbating glycocalyx degradation and ALI.^72^ This modification occurs rapidly in response to metabolic changes and may serve as an early biomarker of lung injury severity. However, as sepsis progresses, the immune response shifts toward an immunosuppressive state, accompanied by distinct epigenetic signatures such as DNA methylation alterations and histone deacetylation that suppress pro-inflammatory gene expression.^74^

Dynamic regulation of epigenetic enzymes further complicates temporal profiling. For example, HDAC3 is upregulated in alveolar epithelial cells during sepsis-induced ALI, promoting mitochondrial dysfunction and epithelial barrier disruption in the early phase, while HDAC3 inhibition in later stages alleviates lung injury by restoring mitochondrial quality control.^108^ Similarly, the methyltransferase EZH2 modulates macrophage polarization and epithelial-mesenchymal transition in a time-dependent manner, influencing both acute inflammation and subsequent fibrosis.^67^ These findings underscore the importance of capturing epigenetic changes at multiple time points to fully understand their roles and therapeutic windows.

Longitudinal studies in septic patients remain scarce but are essential for mapping the temporal dynamics of epigenetic marks. Such investigations would facilitate identification of early predictive markers for risk stratification and late markers indicative of immunosuppression or tissue repair. Furthermore, dynamic epigenetic profiling could guide timing of epigenetic therapies, which may exert opposite effects depending on disease stage. For instance, HDAC inhibitors might be beneficial during hyperinflammation but potentially harmful if administered during immunosuppression.^109^ Technological advances enabling minimally invasive sampling and high-throughput epigenomic analysis facilitate repeated measurements, yet integration of temporal data into clinical decision-making remains challenging. Computational modeling and machine learning approaches may help delineate dynamic epigenetic trajectories and predict optimal intervention points.

#### Confounding factors: Influence of age, comorbidities, and medications on epigenetic patterns; need for adjustment in model construction

The interpretation and clinical application of epigenetic biomarkers in SI-ALI are significantly influenced by numerous confounding factors that affect epigenetic patterns independently of the disease process. Age, comorbidities, and concomitant medications represent the most critical variables that must be considered in biomarker development and predictive model construction to avoid biased or misleading conclusions. Aging is associated with widespread epigenetic alterations, including global DNA hypomethylation, site-specific hypermethylation, and changes in histone modification patterns—collectively termed the “epigenetic clock”.^74^ These age-related changes influence immune cell function and inflammatory responses, potentially confounding epigenetic signatures attributed to sepsis or ALI. For instance, elderly patients may exhibit baseline epigenetic profiles that predispose them to exaggerated inflammatory responses or impaired resolution, thereby affecting disease susceptibility and biomarker readouts.^110^

Comorbidities such as diabetes, chronic lung disease, and cardiovascular disorders also modulate the epigenetic landscape. Chronic inflammation and metabolic dysregulation inherent to these conditions can induce lasting epigenetic alterations in immune cells and structural cells, altering their responses to acute insults like sepsis. For example, metabolic-epigenetic circuits involving histone lactylation have been implicated in sepsis pathogenesis, linking metabolic alterations to transcriptional reprogramming.^73^ Consequently, failure to adjust for comorbidities may confound associations between epigenetic marks and sepsis outcomes. Medications commonly administered in critical care—including sedatives, antibiotics, and corticosteroids—may also influence epigenetic regulation. Certain antibiotics have been shown to affect DNA methylation and histone acetylation, potentially altering immune cell function. Sedatives may influence gene expression through epigenetic mechanisms, further complicating interpretation. Moreover, epigenetic therapies themselves, such as HDAC inhibitors, are being explored for sepsis treatment, underscoring the need to distinguish medication effects from disease-related epigenetic changes.^109^ To address these confounding factors, rigorous study designs incorporating well-matched control groups and comprehensive clinical data are essential. Statistical models must include adjustments for age, comorbidities, and medication exposure to isolate sepsis-specific epigenetic signals. Multi-omics integration and machine learning approaches may facilitate identification of robust biomarkers resistant to confounding influences. Additionally, stratified analyses may reveal subgroup-specific epigenetic patterns, enabling personalized risk stratification and treatment.

## Integrating multi-omics data to construct precision prediction models

### The necessity and strategies of multi-omics integration

The complexity of SI-ALI arises from multifaceted molecular and cellular interactions that cannot be fully captured by any single omics approach alone. Individual omics data types—such as genomics, epigenomics, transcriptomics, proteomics, or metabolomics—provide valuable yet inherently limited snapshots of biological processes. For instance, genomics reveals genetic susceptibility loci, while transcriptomics reflects gene expression changes; proteomics and metabolomics capture functional protein abundance and metabolic alterations, respectively.^111^ However, these layers operate in concert, and isolated analyses may overlook critical interactions and regulatory mechanisms essential for understanding SI-ALI pathogenesis. Therefore, integration of multi-omics data—encompassing genetic susceptibility loci, epigenetic modifications, transcriptomic profiles, proteomic landscapes, and metabolomic signatures—is indispensable for constructing comprehensive molecular maps of SI-ALI. Such integration enables delineation of complex molecular networks, identification of key regulatory nodes, and elucidation of dynamic biological pathways driving disease progression and heterogeneity. For example, multi-omics studies in related fields have demonstrated how combining transcriptomic and metabolomic data can reveal metabolic reprogramming in hypoxic responses, or how integrating proteomic and phosphoproteomic data can uncover signaling cascades in inflammation and tissue injury.^112^

The challenge of integrating such high-dimensional, heterogeneous datasets necessitates advanced computational strategies. Machine learning and artificial intelligence algorithms have emerged as critical tools for managing and interpreting multi-omics data. Techniques including random forests, support vector machines, deep learning, and contrastive learning frameworks enable extraction of meaningful patterns from complex datasets, overcoming issues of noise, redundancy, and batch effects. For instance, deep learning models such as OmniCLIC have demonstrated superior performance in multi-omics integration and classification tasks by learning omics-specific representations and capturing cross-omics correlations, thereby enhancing both accuracy and interpretability.^113^ Similarly, models combining self-attention mechanisms and capsule networks have been employed to dynamically weight features across omics layers, improving cancer subtype classification and biomarker discovery.^114^ Furthermore, the integration process must address technical challenges such as batch effects arising from data generated across different platforms, laboratories, or time points. Strategies like MultiBaC leverage shared data patterns to correct for batch effects across omics types, thereby improving detection of biologically relevant signals.^115^ Utilization of cloud-based bioinformatics platforms and standardized pipelines further streamlines data processing and integration, enabling reproducibility and scalability.^116^ Importantly, AI approaches not only enhance predictive modeling but also contribute to interpretability of multi-omics data by identifying key molecular features and pathways, as demonstrated in studies of cancer heterogeneity and drug sensitivity analysis.^117^^,^^118^

In the specific context of SI-ALI, multi-omics integration combined with AI can unravel the intricate interplay between immune dysregulation, metabolic reprogramming, and epigenetic modifications driving lung injury. For instance, single-cell multi-omics analyses have revealed metabolic shifts in pulmonary endothelial cells mediated by glycolytic reprogramming, implicating the HIF-1/PI3K-Akt axis in endothelial dysfunction during sepsis-associated lung injury.^112^ Integrating these data with transcriptomic and epigenomic profiles could identify patient-specific molecular subtypes and risk stratification markers, enabling targeted therapeutic interventions. Furthermore, AI-driven multi-omics integration supports development of dynamic biomarker systems that incorporate metagenomics, metabolomics, and immunophenotyping to capture the role of the gut-lung axis in sepsis progression and lung injury.^119^

### Development and validation of clinical prediction models

Developing clinical prediction models for sepsis-induced ALI and ARDS requires a comprehensive strategy that integrates clinical variables with multi-omics markers—including epigenetic biomarkers—to enhance accuracy in risk stratification and prognostic prediction. Initial model construction typically utilizes discovery or derivation cohorts, combining clinical data such as oxygenation indices (e.g., PaO2/FiO2 ratio), inflammatory markers (e.g., TNF-α, IL-6), organ dysfunction scores (e.g., APACHE II, SOFA), and demographic factors with molecular markers derived from genomics, transcriptomics, and epigenetics. For instance, transcriptomic analyses have identified differentially expressed genes (DEGs) including BPI, OLFM4, LCN2, CD24, MMP8, and MME, which—when integrated with clinical parameters such as direct lung injury and shock—form robust nomograms predicting ARDS development in septic patients with high accuracy (AUC approximately 0.86).^120^ Similarly, machine learning approaches have been employed to screen gene signatures (e.g., ARHGDIB, ALDH1A1, TACR3, TREM1, PI3) that distinguish sepsis-induced ALI with favorable diagnostic performance (AUC up to 0.83) and identify potential therapeutic targets.^121^ Incorporation of epigenetic biomarkers—such as microRNAs (e.g., hsa-miR-1247-5p) and histone lactylation-related genes (e.g., RBM25)—further refines these models by capturing regulatory mechanisms underlying disease progression and immune dysregulation.^122^ Clinical variables reflecting endothelial dysfunction (e.g., soluble thrombomodulin, vascular cell adhesion molecule-1) have also been combined with clinical data to predict persistent acute respiratory dysfunction in pediatric sepsis, achieving high discrimination (AUC approximately 0.88).^123^ These composite models leverage the synergistic value of clinical and molecular data to stratify patients at risk for developing severe ARDS or mortality, thereby facilitating early identification and targeted intervention.

Following development, models must undergo rigorous validation in independent, prospective, and preferably multi-center cohorts to assess their generalizability, calibration, discrimination, and clinical utility. Validation studies aim to evaluate a model’s ability to accurately predict outcomes such as ARDS development, severity, or mortality across diverse patient populations and clinical settings. For example, a mortality prediction model for pediatric ARDS developed from multinational cohorts demonstrated favorable discrimination (AUC ≥0.82) and was externally validated in independent cohorts, although calibration adjustments may be needed to optimize performance.^124^ Similarly, a nomogram predicting sepsis-associated ALI based on clinical variables (PaCO2, PaO2, serum uric acid, SOFA score) exhibited excellent discrimination (AUC >0.91) and calibration in both training and validation cohorts, with decision curve analysis confirming clinical applicability.^125^ Validation extends to biomarker-based models as well; for instance, a transcriptomic biomarker nomogram for ARDS prediction demonstrated consistent calibration and decision curve benefits, supporting its potential clinical utility. Furthermore, models incorporating endothelial biomarkers have been validated across derivation and test cohorts, maintaining robust AUC values (0.78–0.83) and reproducibility.^123^ These validation efforts underscore the importance of prospective, multi-center data for confirming model robustness, addressing patient population heterogeneity, and ensuring reliability prior to clinical application.

The ultimate goal of developing these composite clinical prediction models is their seamless integration into electronic health record (EHR) systems to provide real-time, actionable decision support for clinicians managing sepsis-induced ALI/ARDS. Embedding predictive algorithms within EHR platforms enables automated risk stratification at the bedside, facilitating timely identification of high-risk patients and guiding personalized treatment strategies. For instance, machine learning-derived models have been developed to identify septic patients at high risk of ALI with high accuracy, and can potentially facilitate early intervention or guide therapeutic strategies.^121^ Additionally, nomograms and risk scores can be translated into user-friendly online calculators or EHR-integrated dashboards, as demonstrated in other critical illness contexts such as inflammatory bowel disease mortality prediction, where online tools enable bedside risk assessment with high accuracy.^126^ Incorporating epigenetic and multi-omics biomarkers into EHR-based models presents challenges related to data standardization, turnaround time, and cost, but advances in rapid molecular diagnostics and bioinformatics pipelines are progressively overcoming these barriers. Real-time integration also supports dynamic risk assessment, enabling clinicians to update prognostic evaluations as new clinical or biomarker data become available, thereby enhancing precision medicine approaches in sepsis management. Ultimately, embedding validated prediction models into EHR systems holds promise for improving clinical outcomes in SI-ALI through personalized risk stratification, optimized resource allocation, and guidance of targeted therapies.

## Future directions and clinical application prospects

### Roadmap from discovery to clinical application

Translating epigenetic biomarkers for SI-ALI from the laboratory to the clinic requires robust, large-scale, prospective, and longitudinal cohort studies to comprehensively capture the dynamically evolving epigenetic landscape throughout disease progression. Current evidence underscores the complexity and heterogeneity of sepsis and its organ-specific manifestations, particularly in the lungs, where immune dysregulation and epigenetic reprogramming play pivotal roles in pathogenesis and prognosis.^63^ However, most existing studies are limited by small sample sizes, cross-sectional designs, or lack of longitudinal follow-up, impeding the ability to discern temporal epigenetic changes associated with disease progression, treatment response, and outcomes. Establishment of international consortia—such as the International Sepsis Epigenomics Initiative—would enable systematic collection of multi-omics data—including DNA methylation, histone modifications, and non-coding RNA profiles—from well-phenotyped cohorts of patients with SI-ALI spanning diverse populations. Such an approach would facilitate identification of reproducible and clinically actionable epigenetic biomarkers reflecting underlying pathophysiological mechanisms, such as lactylation of histone H3K18 and EGR1 promoting endothelial glycocalyx degradation in SI-ALI,^72^ or the RBM25-ACLY axis linking metabolic reprogramming to epigenetic transcriptional regulation.^73^ Longitudinal sampling would also permit monitoring of epigenetic changes in response to interventions and disease evolution, providing insights into mechanisms of immune resilience and tolerance critical for recovery. Furthermore, integrating epigenetic data with clinical phenotypes and other molecular layers—genomics, transcriptomics, proteomics—within large cohorts enables stratification of patients into biologically and clinically meaningful subgroups, overcoming limitations of conventional clinical classifications. Such stratification is essential for precision medicine, as it can reveal distinct epigenetic signatures associated with different SI-ALI endotypes, thereby informing prognosis and guiding targeted therapies.

Adoption of adaptive clinical trial designs—such as umbrella and basket trials—adjusted according to epigenetic stratification represents a transformative strategy for accelerating clinical translation of precision therapies in SI-ALI.^127^ Traditional randomized controlled trials often fail to account for the molecular heterogeneity inherent to sepsis and its complications, leading to inconclusive or negative results despite promising preclinical data. In contrast, adaptive trials incorporating epigenetic biomarkers for patient stratification enable evaluation of targeted interventions within biologically defined subgroups, thereby increasing likelihood of therapeutic efficacy while minimizing unnecessary exposure to ineffective treatments. For instance, patients exhibiting specific epigenetic modifications—such as aberrant DNA methylation patterns or histone lactylation profiles associated with immune dysregulation and lung injury—could be enrolled into distinct trial arms testing agents modulating these epigenetic pathways. The discovery of the SNHG1/DNMT1/miR-495 axis as a regulator of inflammation and pyroptosis in sepsis-associated ALI highlights a potential therapeutic target amenable to epigenetic modulation.^64^ Adaptive trial designs allow real-time modification of trial parameters based on interim analyses, facilitating identification of responders and non-responders and enabling dynamic allocation of patients to the most promising treatment regimens. Umbrella trials focus on testing multiple targeted therapies within a single disease entity stratified by distinct epigenetic markers, while basket trials test a single targeted therapy across multiple diseases or conditions sharing the same epigenetic alteration. Both approaches are particularly suited to SI-ALI, a complex and multifaceted condition where multiple epigenetic mechanisms contribute to pathogenesis. Moreover, integration of epigenetic biomarker assessment into trial protocols necessitates development of rapid, reliable, and standardized assays to guide patient selection and monitor treatment response.

To fully realize the potential of epigenetic biomarkers for risk stratification and targeted therapy in SI-ALI, establishment of international consensus guidelines is essential to standardize biomarker detection methodologies, reporting practices, and data sharing protocols. The field of epigenetics is characterized by diverse analytical platforms, variable sample processing protocols, and heterogeneous data interpretation frameworks, collectively hindering reproducibility and comparability across studies. Standardization efforts should encompass pre-analytical variables—such as sample type (e.g., blood, bronchoalveolar lavage fluid, lung tissue), collection time points relative to disease onset, and storage conditions—all of which can significantly influence epigenetic results. Analytical standardization involves harmonization of detection technologies for DNA methylation (e.g., bisulfite sequencing, methylation arrays), histone modification profiling (e.g., ChIP-seq), and non-coding RNA quantification, ensuring sensitivity, specificity, and inter-laboratory reproducibility. Reporting standards must mandate comprehensive documentation of experimental design, patient demographics, clinical parameters, and bioinformatics pipelines used for data analysis, facilitating transparent interpretation and meta-analysis. Furthermore, creation of centralized, accessible databases adhering to FAIR (Findable, Accessible, Interoperable, Reusable) principles is critical for promoting data sharing and collaborative research. Such repositories would enable pooling of epigenetic datasets from diverse cohorts, facilitating cross-validation of biomarkers and accelerating discovery. International consortia involving clinicians, researchers, regulatory agencies, and industry stakeholders should lead development of these consensus guidelines, drawing on successful models from genomics and proteomics. This collaborative framework will also address ethical considerations related to patient privacy and data security.

### Personalized therapy and dynamic monitoring

The emergence of precision medicine for SI-ALI hinges critically on the ability to rapidly and accurately characterize the epigenetic landscape of individual patients at the earliest stages of disease. This approach envisions deployment of rapid, point-of-care-compatible assays to capture patients’ unique epigenetic signatures—including DNA methylation patterns, histone modifications, and non-coding RNA profiles—thereby stratifying them into distinct risk categories and therapeutic subgroups. Such stratification is essential, as sepsis exhibits heterogeneous immune dysregulation that varies not only between patients but also across affected organs including the lungs, heart, liver, and kidneys. For instance, epigenetic reprogramming events such as histone lactylation—driven by metabolic shifts during sepsis—have been implicated in modulating immune responses and organ-specific injury pathways. Identification of lactylation-related gene signatures, including key regulators such as RBM25 and ACLY, provides a promising molecular foundation for prognostic stratification and targeted intervention in SI-ALI.^73^ By integrating these epigenetic biomarkers into rapid diagnostic platforms, clinicians can assign patients to customized treatment regimens that may include specific epigenetic modulators—such as HDAC inhibitors or DNMT inhibitors—or immunomodulators targeting cytokine pathways or regulatory T cell function. This precision approach aims to optimize therapeutic efficacy while minimizing off-target effects and systemic toxicity, thereby addressing the challenges posed by “one-size-fits-all” approaches in current sepsis management. Furthermore, the compartmentalized nature of immune responses in sepsis underscores the need for organ-specific epigenetic profiling to guide localized interventions, such as targeting the Spns2/S1P axis in pulmonary macrophages to mitigate lung inflammation and injury.

Dynamic monitoring of epigenetic biomarkers throughout the disease course of SI-ALI represents a critical step toward real-time, adaptive precision medicine. Unlike static baseline measurements, longitudinal assessment of epigenetic modifications—such as changes in histone lactylation levels, DNA methylation alterations, or circulating microRNA profiles—enables continuous insight into a patient’s evolving immune and metabolic status. This dynamic monitoring allows clinicians to promptly evaluate the effectiveness of administered therapies and detect early signs of treatment resistance or adverse effects. For example, the RBM25-ACLY axis—linking metabolic alterations to histone lactylation and transcriptional reprogramming—serves not only as a prognostic marker but also as a measurable target for monitoring therapeutic efficacy in sepsis.^73^ By tracking fluctuations in this axis, clinicians could adjust dosing of epigenetic drugs or switch immunomodulatory strategies based on real-time molecular feedback. Similarly, monitoring epigenetic markers associated with immune resilience—such as the epigenetic status of regulatory T cells or methylation of cytokine gene promoters—could inform modulation of immune responses to prevent either hyperinflammation or immunosuppression, both of which contribute to adverse outcomes in sepsis.^63^ Furthermore, circulating epigenetic biomarkers, including microRNAs associated with ARDS, provide minimally invasive means for assessing lung injury progression and recovery, facilitating timely treatment adjustments.^128^ Implementation of such dynamic epigenetic monitoring necessitates integration with advanced bioinformatics and machine learning algorithms capable of interpreting complex time-series data to guide clinical decision-making. This approach holds promise for overcoming limitations of static risk assessment and empirical treatment, enabling a truly responsive, individualized management paradigm.

## Conclusion

The exploration of epigenetic biomarkers in SI-ALI has substantially advanced our molecular understanding of this complex and heterogeneous disease. From an expert perspective, the integration of epigenetic insights into clinical practice represents a transformative shift toward precision medicine, offering refined approaches for risk stratification, prognostic assessment, and therapeutic guidance. This review has highlighted the multifaceted roles of DNA methylation, non-coding RNAs, and histone modifications as dynamic and sensitive indicators of disease subtypes, progression, and outcomes. Their capacity to capture the spatiotemporal heterogeneity inherent to SI-ALI underscores their value not only as diagnostic and prognostic tools but also as real-time monitors of disease evolution.

Weighing the evidence across studies, it becomes evident that epigenetic modifications provide a robust framework for deciphering the molecular basis of SI-ALI heterogeneity. DNA methylation patterns have emerged as stable yet adaptable markers reflecting both genetic predisposition and environmental influences; non-coding RNAs, particularly microRNAs, offer a versatile regulatory layer fine-tuning the inflammatory and immune responses central to SI-ALI pathogenesis; histone modifications further complement this picture by modulating chromatin accessibility and gene expression following septic insult. Collectively, these epigenetic mechanisms form an interconnected network that not only illuminates disease mechanisms but also identifies actionable targets for intervention.

The therapeutic implications of these findings are profound. Epigenetic-targeted therapies—including the use of epigenetic drugs and miRNA modulators—represent promising strategies for directly influencing the pathological processes driving SI-ALI. However, translation of these approaches from bench to bedside remains in its early stages. Preclinical models have demonstrated efficacy in attenuating lung injury and inflammation through modulation of key epigenetic regulators, yet clinical validation is constrained by challenges including patient population heterogeneity, variability in epigenetic profiles, and complexities in dosing and delivery mechanisms. This necessitates rigorous clinical trial designs incorporating standardized epigenetic assessment protocols and stratified patient cohorts to accurately evaluate therapeutic efficacy and safety.

Moreover, the path to successful clinical implementation is complicated by technical and methodological hurdles. Standardization of epigenetic biomarker assays and quantification is essential to ensure reproducibility and comparability across studies. Validated animal and cellular models that faithfully recapitulate the epigenetic landscape of human SI-ALI are indispensable for mechanistic studies and preclinical testing. Additionally, clinical trial designs must incorporate adaptive frameworks that accommodate the dynamic nature of epigenetic changes and allow for personalized treatment adjustments. Addressing these challenges requires interdisciplinary collaboration among molecular biologists, clinicians, bioinformaticians, and biostatisticians, supported by large-scale, well-phenotyped patient cohorts to generate high-quality, generalizable data.

Looking forward, integration of epigenetic data with other omics layers—such as genomics, transcriptomics, proteomics, and metabolomics—is pivotal for constructing comprehensive, multi-dimensional precision medicine models for SI-ALI. Application of artificial intelligence and machine learning algorithms to these complex datasets can facilitate identification of novel biomarkers, predictive signatures, and therapeutic targets with unprecedented accuracy. Such integrative approaches hold promise for revolutionizing clinical decision-making through enabling early diagnosis, individualized risk assessment, and tailored therapeutic interventions that dynamically adjust as the disease progresses.

In summary, epigenetic biomarkers have emerged as pivotal tools for unraveling the heterogeneity of SI-ALI and hold immense potential for reshaping its clinical management. While substantial progress has been made in characterizing these molecular features and exploring targeted therapies, the field must overcome significant translational barriers through standardized methodologies, robust validation, and interdisciplinary collaboration. The future of SI-ALI management lies at the convergence of epigenetics with multi-omics and artificial intelligence, paving the way toward truly personalized medicine that improves patient outcomes and reduces the global burden of this devastating condition. As research continues to evolve, sustained efforts to bridge basic science and clinical application will be essential to fully realize the potential of epigenetic precision medicine in SI-ALI.

## Acknowledgments

The authors would like to thank all researchers who shared their data publicly and made this project possible. Funding This work was supported by the Medical Research Fund Project of Zhongshan City (grant no. 20261A010391).

## Author contributions

J.W.: writing – original draft, methodology, resources, formal analysis, visualization, writing – review and editing. R.X. and J.C.: writing – original draft, conceptualization, investigation. Y.Z., Z.D., and X.C.: writing – original draft, formal analysis, validation. Y.H., Z.H., and C.P.: formal analysis, supervision, funding acquisition, conceptualization, writing – review and editing.

## Declaration of interests

The authors declare no competing interests.
